# A neural classification method for supporting the creation of BioVerbNet

**DOI:** 10.1186/s13326-018-0193-x

**Published:** 2019-01-18

**Authors:** Billy Chiu, Olga Majewska, Sampo Pyysalo, Laura Wey, Ulla Stenius, Anna Korhonen, Martha Palmer

**Affiliations:** 1 Language Technology Laboratory, MML, University of Cambridge, 9 West Road, Cambridge, CB39DB UK; 2 Department of Biochemistry, University of Cambridge, Cambridge, CB2 1QW UK; 3 Institute of Environmental Medicine, Karolinska Institutet, Stockholm, 210-171-77 Sweden; 4 Department of Linguistics, University of Colorado at Boulder, Colorado, 80309-0295 USA

**Keywords:** Verb lexicon, Representation learning

## Abstract

**Background:**

VerbNet, an extensive computational verb lexicon for English, has proved useful for supporting a wide range of Natural Language Processing tasks requiring information about the behaviour and meaning of verbs. Biomedical text processing and mining could benefit from a similar resource. We take the first step towards the development of BioVerbNet: A VerbNet specifically aimed at describing verbs in the area of biomedicine. Because VerbNet-style classification is extremely time consuming, we start from a small manual classification of biomedical verbs and apply a state-of-the-art neural representation model, specifically developed for class-based optimization, to expand the classification with new verbs, using all the PubMed abstracts and the full articles in the PubMed Central Open Access subset as data.

**Results:**

Direct evaluation of the resulting classification against BioSimVerb (verb similarity judgement data in biomedicine) shows promising results when representation learning is performed using verb class-based contexts. Human validation by linguists and biologists reveals that the automatically expanded classification is highly accurate. Including novel, valid member verbs and classes, our method can be used to facilitate cost-effective development of BioVerbNet.

**Conclusion:**

This work constitutes the first effort on applying a state-of-the-art architecture for neural representation learning to biomedical verb classification. While we discuss future optimization of the method, our promising results suggest that the automatic classification released with this article can be used to readily support application tasks in biomedicine.

**Electronic supplementary material:**

The online version of this article (10.1186/s13326-018-0193-x) contains supplementary material, which is available to authorized users.

## Background

Natural Language Processing (NLP) and text mining of biomedical literature are critically important for the management of rapidly growing literature in biomedical sciences. Core bio-NLP technologies such as syntactic and semantic parsing, event identification, relation extraction, and entailment detection can all benefit from rich computational lexicons containing information about the behaviour and meaning of words in biomedical texts. While relatively well-developed resources are available for nouns in biomedicine (e.g. UMLS Metathesaurus, [1]), verb-related resources are still lacking in both depth and coverage [2–6].

One particularly useful verb resource for general domain NLP is VerbNet [7]. Providing detailed syntactic and semantic information for English verbs, this broad-coverage resource has proved useful in supporting a wide variety of NLP tasks and applications, including word sense disambiguation [8], semantic role labelling [9], semantic parsing [10], information extraction [11] and text mining applications [12, 13], among others.

Our ultimate aim is to create BioVerbNet – the first VerbNet for supporting NLP and text mining in biomedicine. However, because manual VerbNet-style classification is a highly expensive and time-consuming task, we first investigate a data-driven approach to the creation of this resource. Previous works have shown that while an unsupervised verb clustering approach based on conventional NLP has the advantage of discovering novel verb classes from corpus data with minimal prior knowledge, such automatically acquired classes necessarily contain quite a lot of noise [14]. Conversely, when training data is available, supervised verb classification can yield higher precision. Korhonen et al. (2006) manually developed a VerbNet-style gold standard for evaluation of automatic verb classification in biomedicine [15] (detailed in “Automatic verb classification” section). We take this resource as a starting point in supervised classification aimed at finding novel member verbs and classes in data, with the idea that human evaluators can validate the output and use the correct classifications as a starting point for the development of BioVerbNet.

Most existing methods for automatic verb classification rely heavily on feature engineering, which is time-consuming and requires expert knowledge [16]. Hence, we automate the process of feature learning by using a neural learning approach, followed by the application of the Nearest Centroid Classifier to assign verbs into classes. We encode word features into a low-dimensional space using neural networks [17–19]. Neural word representations (embeddings) serve now as invaluable features in a broad range of NLP tasks, including named entity recognition [20–22] and text classification [23, 24]. Neural representation models such as the skip-gram model with negative sampling (SGNS) are highly efficient in capturing syntactic and semantic properties of words in corpora and are therefore intuitively useful also for VerbNet-style classification [25].

Our methodology consists of two steps: First, we apply the recent method by Vulić et al. [26] to identify best contexts for learning biomedical verb representations. The method, based on the skip-gram model with negative sampling (SGNS), has produced successful results in the general domain but has not previously been applied to specialised domains such as biomedicine. It involves first creating a context configuration space based on dependency relations between words, followed by applying an adapted beam search algorithm to search this space for the class-specific contexts, and finally using these contexts to create class-specific representations.

In this work, we apply the method to a large biomedical corpus: the PubMed Central Open Access subset [27] and all the PubMed abstracts, consisting of about 10 billions tokens and 27 million word types in total. We evaluate the trained representations against a gold standard aimed at capturing verb similarity in biomedicine (BioSimVerb, [6]). Our results show that when the model is optimized with context configuration for verbs, it outperforms the baseline model (a standard SGNS without verb-specific contexts) significantly, yielding a 5 point improvement in Spearman’s rank correlation (referred to as *ρ* henceforth).

In the second step, the optimized representation is used to provide word features for building a verb classification. This is obtained by expanding the small manually developed VerbNet-style classification of 192 biomedical verbs by Korhonen et al. [15] (details in “Automatic verb classification” section) with 957 new candidate verbs. The candidate verbs are chosen from BioSimVerb (details in “Verb classification” section), based on their frequent occurrence in biomedical journals across 120 subdomains of biomedicine (as categorized by Broad Subject Terms [28]). This ensures the wide coverage of verb classification ideal for the development of BioVerbNet. We use the Nearest Centroid Classifier to connect the new candidates to an appropriate class in the resource of Korhonen et al. [15]. The resulting classification provides 1149 verbs assigned to the 50 classes in the original resource. It lists, for each verb, the most frequent dependency contexts that reflect their syntactic behaviour along with example sentences.

Qualitative evaluation of the automatically expanded classes by linguists and biologists reveals that the method is highly accurate: the vast majority of the novel members verbs and classes are legitimate. The method can therefore be used to greatly facilitate the development of BioVerbNet by hypothesizing novel classifications for expert validation. We discuss further optimization of the method for real-life computational lexicography, but our promising results suggest that the automatic classification released with this article can be used to readily support NLP application tasks in biomedicine.

Apart from proposing an automatic approach to the creation of BioVerbNet, our study provides an investigation of how different types of dependency-based contexts influence the learning of verb representations in biomedicine. The optimal context configuration proved to be highly domain-specific. Our results and insights can facilitate researchers to develop useful methods for training class-specific representations for biomedical NLP. The resources are publicly available to the research community under an Open Data license at: https://github.com/cambridgeltl/bio-verbnet.

## Related work

### Computational verb lexicons

VerbNet [7] is the most extensive verb lexicon currently available in the general domain. It consists of verbs grouped into classes based on their shared syntactic and semantic properties, such as syntactic frames, semantic roles of arguments, etc. For example, the members (e.g. *delete* and *discharge*) in the verb class *Remove* have similar frames and meaning, and can be used to describe similar events. VerbNet classes have supported many NLP tasks, such as word sense disambiguation [8], information extraction [11] and text mining applications [12, 13]. The current version of VerbNet (v3.3) consists of 9344 verbs organised in 329 main classes [29]. Although it has a wide coverage for general domain NLP applications, it is not designed for specialized domains, such as biomedicine, where verbs tend to have a very different meaning and behaviour than in general English [2, 3]. Hence, there is a need to develop domain-specific resources to support biomedical NLP.

Some large lexical resources, such as UMLS Metathesaurus [1], can be found in the biomedical domain. However, most of them focus on nouns and do not provide a good coverage of other important word classes like verbs. The lexicons which cover biomedical verbs are usually smaller in scale and limited to certain sub-domains in biomedicine. For example, the UMLS SPECIALIST lexicon [30], which is created manually by lexicographers, mainly contains medical and health-related vocabularies. On the other hand, the BioLexicon [31] – a corpus-driven lexicon which contains syntactic and semantic frame information for verbs – is extracted from the Escherichia Coli (E.Coli) domain, which limits its usefulness to applications that deal with other sub-domains of biomedicine.

### Automatic verb classification

Verb classification links together syntactic and semantic properties of groups of verbs by means of lexical classes. Such grouping can reduce the parameters used for representing verbs individually. While it is time-consuming to manually classify a large number of verbs, previous studies have shown that it is possible to automatically acquire verb classes from both general [32–35] and biomedical texts [15, 36, 37]. For example, Li and Brew (2008) classify 1,300 verbs into 48 Levin classes using Bayesian Multinomial Regression for classification [38]. A range of verb features have been explored in their works, including the dependency relations between the arguments and the prepositions. On the other hand, Sun (2013) uses rich features based on the predicate-argument structure (e.g. verb subcategorization frames and selectional preferences) to classify 192 biomedical verbs into 50 classes [37]. From the cognitive science perspective, Barak et al. (2014) apply a two-stage Bayesian model to cluster verbs (first based on syntax then on semantic classes) in order to analyze how computational clustering is similar to human verb knowledge generalization [35]. Apart from these, methods which induce verb classes of other languages (e.g. Estonian [39] and German [40]) as well as of a particular type of verb (e.g. Propositional attitude verbs including *think* and *want* [41]) have also recently emerged.

Both supervised and unsupervised approaches have been proposed for verb classification: A supervised approach assigns verbs into one of several pre-defined verb classes, whereas an unsupervised approach uses clustering techniques to induce verb classes based on similarity between verbs. The two types of approaches can serve different purposes: An unsupervised approach requires less prior knowledge and can be used to discover new classes in scenarios where no manually created classification (i.e. training data) is available; however, the resulting classification unavoidably contains noise. In contrast, when relevant training data is available, supervised approaches have an immediate advantage in terms of precision of the verbs they classified, as reflected in previous studies [42]. For example, Sun et al. (2008) classify 204 verbs into 17 Levin classes, using three supervised classifiers (Support Vector Machines, Maximum Entropy and Gaussian method) and one unsupervised method (Pairwise clustering) [42]. They report a better result when using the supervised method (Gaussian) and a markedly worse result when using the unsupervised method (pairwise clustering). Hence, the supervised approach can be useful for supplementing existing classification with additional (and more accurate) members when training data is available. In this regard, Korhonen et al. (2006) manually developed a VerbNet-style gold standard for verb classification in biomedicine (Table 1), containing 192 verbs organised into a class taxonomy consisting of 50 fine-grained classes for biomedical verbs [15]. To the best of our knowledge, it is the only biomedical resource of this type. We use this resource as a starting point for creation of a supervised approach intended to facilitate the development of BioVerbNet. Our basic idea is to expand the resource by automatically connecting new candidate verbs to existing verbs based on their euclidean distance found in the vector space of an optimized representation model (see the details in “Verb classification” section).

**Table 1:** Example gold standard classes and class members from Korhonen et al. (2006) [15]

Index	Class name	Subclass name	Example members
2.2.1	Biochemical events	Biochemical modification	dephosphorylate, phosphorylate
4.1.3	Experimental procedure	Label	stain, label, immunoblot, probe, fix
4.2.0		Precipitate	coprecipitate, coimmunoprecipitate, precipitate
9.1.1	Report	Examine	assess, evaluate, estimate, examine, explore, analyze
9.1.2		Establish	establish, test, investigate
9.2.1		Presentational	argue, hypothesize, conclude, reason, note, speculate, assume
10.1.1	Perform	Quantitate	quantify, quantitate, measure, monitor
11.0.0	Release	Release	release, detach, excise, dissociate
12.0.0	Use	Use	utilize, employ, exploit
14.0.0	Call	Call	name, designate
16.0.0	Appear	Appear	become, occur, seem

A vast majority of previous works in automatic verb classification rely heavily on feature engineering. This is a time-consuming and expensive approach that does not port easily to new tasks, and therefore does not provide an optimal solution for classification of verbs in specific domains. Works which perform verb classification on automatically-learned features (through neural networks) are emerging recently. For example, Vulić et al. (2017) perform verb classification across multiple languages based on automatically-learned features [26]. These sets of features are unsupervisedly induced from corpora (without expert knowledge or feature engineering). They report state-of-the-art results in verb classification across six languages as compared with previous studies that extract features using complicated language-specific resources. In this paper, we address this problem and introduce an approach that combines the benefit of supervision with that of automatic feature learning using neural networks.

### Representation learning

We base our feature learning on representation learning. In recent past there have been a lot of work on encoding word features into a low-dimensional space in an unsupervised fashion using artificial neural networks [17–19]. These representation models encode the linguistic properties of words in a form where semantically similar words appear closely in vector space. They have proved highly popular and successful for many NLP tasks, including named entity recognition [20–22, 43], event identification [44], relation extraction [45] and text classification [23, 24]. Among them, the skip-gram model with negative sampling (SGNS, [46]) has achieved cutting-edge results in a range of semantic tasks such as sentence completion and analogy [46, 47].

In the original SGNS, the representation of a word is learned by predicting all its neighbouring words in a window (contexts), assuming all contexts are useful. However, some contexts that are useful for the representation learning of one word class may be uninformative for another one. For example, a noun pre-modifier may be useful for learning noun representations but not verb representations. Hence, other types of contexts, such as dependency relations and symmetric patterns (e.g. *x* and *y*), have been proposed [48, 49]. These studies show that the quality of specific word class representation (e.g. nouns or verbs) is intrinsically linked to the contexts they learned from. For example, Schwartz et al. (2015) report that symmetric patterns (e.g. *x* or *y*) are essential as contexts for verb and adjective representations whereas BOW is useful for noun representation [48]. In the general domain, Vulić et al. (2017) propose a framework for identifying the most useful (class-specific) contexts for learning representations for nouns, verbs and adjectives, respectively [26]. Such class-specific optimization can greatly extend the usefulness of representation models for tasks relating to a particular word class.

However, all these studies have only involved general domain text, and their results do not necessarily apply to biomedical text. In our work, we aim to identify the optimal dependency-based context configurations for learning representations of biomedical verbs whose lexical characteristics can be distinct from general domain verbs. We adopt the recent framework of Vulić et al. to perform class-specific optimization for biomedical verb representation. In this framework, a context configuration space is created based on the dependency relations between words, followed by using an adapted beam search algorithm to search this space for the class-specific contexts. These contexts are then used to create class-specific representations. The authors show that selecting class-specific contexts helps representation models better capture the semantic and syntactic properties for individual word class, which renders the technique particularly useful for our purposes. We also extend the usefulness of the framework by using our optimized representation to create a new verb classification for biomedicine. In the next section, we will describe our implemented framework for context selection.

## Dataset design

### Context selection

Our aim is to fine-tune the learning of verb representation so that it can be used to build a verb lexicon. For this, we first identify contexts contributing to biomedical verb representation learning. We use the Stanford typed dependencies (DEPS, [50]) as contexts for selection. This is because, first, DEPS can help representation models learn lexical information beyond the BOW context window and, second, they can provide a natural grouping of related words [26]. For example: (*contain, glucose_dobj*) and (*generates, radiation_dobj*) which share the same dependency *dobj* can be grouped into the *dobj* bag (referred to as context bag henceforth). In the next section, we describe how we construct these context bags.

#### Creation of context bags

We organised the dependency-parsed corpus for training representation in the form of (*word, context*) pairs, as in the work of Levy and Goldberg [49]. *Word* is the target word for training the representation model, whereas *context* stands for its corresponding context elements in text (e.g. dependency relations and the head of the dependent word). To give an example, the pair (*modulator, efficient_amod*) denotes a target word *modulator* with an adjectival modifier (*amod*) context: *efficient*. Given the dependency-parsed corpus, we break it down into individual context bags based on the dependency relation of each (*word, context*) pair. Hence, the context bag *dobj* consists of pairs such as *(regulate, cells_dobj)* or *(fire, neuron_dobj)*. We follow the same procedure as Vulić et al. to process the context bags. First, Prepositional and Conjunction relations are collapsed. Hence, all pairs with (*prep_x*) or (*conj_y*) such as (*prep_in*) and (*conj_or*) will be merged into the context bags (*prep*) and (*conj*) correspondingly. Second, similar dependencies (i.e. those at the bottom two levels of each dependency type in the Stanford dependency hierarchy) are merged. For example, direct (*dobj*) and indirect objects (*iobj*) are merged into the context bag (*obj*). Third, infrequent pairs and uninformative dependencies are removed (e.g., *punctuation*). A *context configuration* denotes a set of individual context bags used for training representation models. We call a configuration consisting of *M* individual context bags a *M-set configuration*. Examining every possible context configuration is computationally expensive when there are many context bags. For example, assessing all contexts in a 10-set configuration (i.e. 10 context bags) would involve training 2 ^10^−1 = 1023 different representation models. We aim to improve the representation without exhaustively evaluating all possible combinations. To achieve this, we apply the context selection framework proposed by Vulić et al., which uses a beam search [51] style selection to reduce the numbers of visited configurations. We will describe the details in the next section.

#### Configuration search

We implement the framework for context selection as proposed by Vulić et al. First, we filter contexts that are uninformative for learning verb representation. For example, the *nn* bag denotes contexts linked from a noun to its noun pre-modifier. This is likely to be useful for learning noun representations, but not verb representations. Hence, when we evaluate the quality of verb representation trained solely with the *nn* bag, we expect that its score will be low. To filter uninformative contexts, we first train a set of representation models with every context bag we obtained from the dependency-parsed corpus, and evaluate them individually with the Bio-SimVerb dataset, a verb similarity gold standard recently created by Chiu et al. [6] (details are described in “Representation learning” section). A threshold score of *ρ*≥ 0.2 is used to filter uninformative contexts. Consequently, we use 7 context bags as the initial configuration in our experiments. They are: *comp*, *conj*, *prep*, *pcomp*, *rel*, *subj* and *obj*. Vulić et al. suggest this step can effectively remove less relevant contexts at a minimal cost for accuracy [26].

After constructing the initial context configuration, the search algorithm starts from the full *M*-set configuration and tests *M(M-1)*-set configurations in which one individual bag is removed at a time to generate each such configuration. The algorithm narrows down the search by keeping only those sets of configurations which outperform the origin M-set configuration. It continues searching over lower-level *(M-1)*-set configurations until it reaches the lowest level or when no further improvement over its original configurations is found.

Using this context selection framework, the search for the optimal configuration for verbs is reduced to only 27 context configurations out of 127 possible configurations (2 ^7^−1 = 127). This includes seven 1-set configurations (i.e. individual context bag) plus twenty other configurations. After we identify the optimal context configuration for verbs, we train a representation model with this configuration. This model will be used for constructing an initial candidate grouping for our BioVerbNet-style verb classification. We describe our construction in the next section.

### Verb classification

As described earlier, we expand the VerbNet-style classification of biomedical verbs of Korhonen et al. [15] (see “Automatic verb classification” section) with a list of new candidate verbs selected from BioSimVerb (see “Representation learning” section). We use BioSimVerb as a source for candidate verbs for multiple reasons: First, it contains verbs that have been manually validated by domain experts, chosen based on their common usages in biomedical text. This avoids the problem of including overly general verbs such as ’have’ and ’be’ or too specific verbs such as ’x-ray’. Second, these verbs have been sourced from journals across 120 sub-domains of biomedicine (as categorized by Broad Subject Terms [28]), ensuring a wide coverage over different areas. Such wide coverage is essential as our methodology is ultimately aimed at suporting the creation of BioVerbNet. Furthermore, since we evaluate our models against BioSimVerb, we expect that our optimized model can best capture the syntactic and semantic properties of verbs in BioSimVerb. Finally, to connect new candidates to a class in the existing verb resource, we use the Nearest Centroid Classifier. It represents each class by the centroid of its member verbs in vector space (from our optimized representation) and connects the new candidates to their nearest class centroids (in terms of euclidean distance).

### Human evaluation of verb classes

Since our aim is to investigate the suitability of our classification methodology for facilitating the creation of BioVerbNet, we use human experts (two linguists and two biologists) to evaluate the novel member verbs and possible novel classes in the sample of a classifier output. Following well-established practices in related works [37, 52], the task of the experts is to determine whether the new member verbs within each verb class are similar enough in terms of their meaning and syntactic patterns to the existing verbs in the original classification to be legitimate members of the class. Whenever this is the case, the method has accurately learned correct classification. When this is not the case, the verbs are examined further for potential discovery of new subclasses to be included in the original classification. When verbs are clearly misclassified, they are excluded or re-assigned to other classes as agreed by the experts.

For this evaluation, the experts followed guidelines specifically developed for the purpose (can be found in Additional file 1). The data provided for the experts includes the original class names and member verbs from the resource of Korhonen et al. [15], the new member verbs from the classifier output and the set of 10 most frequent dependency contexts for each verb. For example, all verbs from the Class 1.2 are labelled ’Verb of affect’, the class consists of member verbs such as *modulate* and *regulate* which are used to describe events that have an effect on entities. The dependency context of *regulate* as in the sentence *Dox could effectively regulate bFGF expression* is denoted as (*subj#obj*). Thirty sentence examples, three per the ten most frequent dependencies of each verb, are also provided along with the dependency information to demonstrate how each individual verb is used in context.

## Dataset construction

### Data

The dependency-parsed corpus is compiled from the pre-processed PubMed Central Open Access subset (PMC) and PubMed abstracts, which are distributed by Hakala et al. [53]. POS tags and tokens in this resource are generated using the BLLIP constituency parser [54] trained on a biomedical corpus [55]. The resource covers over 26M abstracts and 1.4M full articles with more than 388M parsed sentences. We filter out words that appear fewer than 100 times in text, as suggested in the work of Levy and Goldberg [49]. Consequently, the corpus consists of approximately 27 million word types.

### Word representation models

In this experiment, we use the popular SGNS architecture to train the word representations. Levy and Goldberg have developed a tool which allows SGNS to learn representations from dependency-parsed contexts formatted as (*word, context*) pairs [49]. All representation models used in this experiment are trained with vector dimension (*d=300*). Similar settings can be found in other studies [48, 56]. The baseline we used is a SGNS model trained with all dependency contexts in the corpus (**DEP-ALL**), a SGNS model trained only with the seven verb-related contexts (**POOL-ALL**) we identified in “Configuration search” section (i.e. contexts with evaluation scores *ρ*≥ 0.2 on BioSimVerb) and a standard SGNS trained with bag-of-words contexts (**BOW**) using the *word2vec* tool [46]. They are used to compare against other representation models with different context configurations. The best-performing model (evaluated with BioSimVerb as described in “Representation learning” section) is then used to build the prototype of BioVerbNet that is validated and corrected manually.

### Evaluation of representation models

The BioSimVerb (a word similarity evaluation dataset) is used as the gold standard to measure the quality of our verb representation models. It consists of 1000 verb pairs whose degree of similarity has been ranked by human judges. The similarity ranking for a representation model is computed as the cosine similarity of the vectors of these verb pairs. Following the standard evaluation protocol, we compare the similarity rankings produced by humans and by individual model on those 1,000 verb pairs using the Spearman’s *ρ* correlation. A higher correlation value implies a better model in capturing verb semantics in text.

## Utility and discussion

### Representation learning

We examine whether different context configurations can improve the quality of verb representation when evaluated against human judgements on a verb similarity task (BioSimVerb, as measured in *ρ* points). Results are shown in Table 2. In general, selecting an optimal context configuration for verbs gives better performance. From Table 2, there is an apparent difference (5 *ρ* points) between models trained with and without context selection: While an evident improvement (4 *ρ* points) can already be found when we pool only contexts that are useful for verbs (POOL-ALL, detail in “Word representation models” section) from the generic corpus (DEP-ALL), a further selection among these verb-related contexts yields additional improvements (1 *ρ* point). Overall, the model trained with the best context configuration is approximately 2 *ρ* points over the best baseline. The results indicate that not all contexts contribute to the learning of biomedical verb representations. Hence, identifying verb-specific contexts is valuable for learning verb representations.

**Table 2:** Performance on the BioSimVerb (in *ρ*) using representations learned with different context configurations. (Bold: best-performing configuration and its score)

Baseline	Spearman’s *ρ*
BOW (win=5)	0.4664
DEP-ALL	0.4323
Configurations: Verb	
POOL-ALL	0.4724
conj+obj+pcomp+prep+rel+subj	0.475
conj+obj+prep+rel+subj**(Best)**	**0.4889**
conj+obj+pcomp+prep+subj	0.4578
conj+obj+pcomp+rel+subj	0.4478
conj+obj+pcomp+prep+rel	0.4406
conj+obj+prep+subj	0.4611
conj+obj+rel+subj	0.4572
conj+obj+prep+rel	0.442
comp+obj+pcomp+prep+rel+subj	0.4376
comp+conj+obj+prep+rel+subj	0.4762
comp+conj+obj+pcomp+prep+subj	0.4655
comp+conj+obj+pcomp+rel+subj	0.4583
comp+conj+obj+pcomp+prep+rel	0.4413
comp+conj+obj+prep+subj	0.4635
comp+conj+obj+rel+subj	0.4592
comp+conj+obj+prep+rel	0.442
obj+pcomp+prep+rel+subj	0.4446
obj+prep+rel+subj	0.441

BOW denotes a basic SGNS learned with bag-of-words context with context window size 5. DEP-ALL denotes a configuration where no filtering of context is used. POOL-ALL denotes a configuration where all individual context bags from the verb-related pools are used. “Best” identifies the best-performing configuration found

### Automatic verb classification

To classify verbs into semantic groups, the Nearest Centroid classification is run on top of the class-specific representations, using vector dimensions as features for learning verb classes. The classifier is first trained using the resource from Korhonen et al. (details in “Automatic verb classification” section). It then connects new verbs to classes based on their euclidean distance (details in “Verb classification” section). Consequently, 957 verbs are classified into 50 classes.

### Human validation of verb classes

In order to evaluate the output of the classifier we employed four experts, two linguists and two biologists with at least a postgraduate level of training in their subject areas. The experts first performed the validation of selected classes individually according to the guidelines (included as Additional file 1), and then consulted and discussed their validations in each domain-specific pair and in linguist-biologist pairs. The 14 classes selected for validation were chosen at random from the classifier output, so as to ensure that both the biomedical and the general scientific domains were represented, with 7 classes chosen per domain, each class consisting of 4-28 member verbs.

The experts were presented with written guidelines and the following materials: (1) a file including the verb classes, their original members from Korhonen et al. [15] and the new candidates to be reviewed (Table 3); (2) an Excel spreadsheet for recording the updated index of the class for each verb based on the manual revision of the class candidates, (3) 30 example sentences drawn from the corpus used in the experiment representing the 10 most frequent dependency contexts for each verb. The guidelines instructed the experts to verify whether the new candidate verbs were similar in terms of their meaning as well as syntactic patterns to the existing member verbs in the original classification. The 30 example sentences provided were meant to facilitate the review process by illustrating how a given verb is used in biomedical texts (keeping in mind that this may differ from its typical usage in the general language domain), i.e. the most common syntactic structures in which it appears. Based on the analysis of the semantic and syntactic behaviour of the new candidates with respect to the existing class members, the experts were asked to decide if each new candidate has been correctly assigned to a given class, or if not, whether it should be (a) reassigned to another class in the classification, (b) form a subclass within a broader existing class, or (c) should be moved to a new class altogether (along with some other misclassified verbs); or otherwise, if no appropriate class could be thought of, (d) whether it should be discarded as noise (i.e. a mistake by the classifier). Importantly, a given verb could only be assigned to a single class or subclass (i.e. soft clustering was not permitted).

**Table 3:** Example classes validated by experts

Index	Subclass name	Example members	New candidates
1.1.2	Suppress	suppress, repress	downregulate, transactivate
7.1.0	Collect	harvest, select, collect	decide, pick, cultivate, procure, gather, choose, transfuse, prioritize, obtain
13.1.0	Encompass	encompass, possess, comprise, bear, span, harbor	overlie, display, hold, exhibit, cover, infest, belong, range
14.0.0	Call	call, name, designate	qualify, regard, rename, mention, request
1.4.0	Modify	modify, catalyze	hydroxylate, hydrolyze, methylate, deaminate, esterify, oxidize, detoxify, metabolize
4.1.3	Label	stain, label, immunoblot, probe, fix	supershift, assay, immunostain, tag, immunolabel, clone, postfix, digest, clamp, counterstain, buffer, electroblot, fluoresce, radiolabel, blot
11.0.0	Release	release, detach, excise, dissociate	reinsert, retract, disassemble, deacylate, extrude, remove, depolymerize, mobilize, lose, resect, separate
10.1.3	Conduct	perform, conduct	execute, undertake

### Qualitative analysis

After having completed the validation task, the experts compared and discussed their analyses to come up with the final classification that they agreed on. The results of the validation are presented in Table 4.

**Table 4:** Results of class validation by experts, for seven general scientific (General) and seven biomedical classes (Biomedical), and across the two domains (Total). Bold: the total no of correct/incorrect candidates (in %) as rated by annotators of each sub-group, and the sum of the two

	No. of new candidates	No. of correct candidates	% correct candidates	No. of incorrect candidates	% incorrect candidates
7.1.0 COLLECT	9	6	66.7	3	33.3
9.1.1 EXAMINE	21	19	90.5	2	9.5
9.3.0 INDICATE	11	10	90.9	1	9.1
10.1.3 CONDUCT	2	2	100	0	0.0
13.1.0 ENCOMPASS	8	6	75.0	2	25.0
14.0.0 CALL	5	4	80.0	1	20.0
16.0.0 APPEAR	19	16	84.2	3	15.8
**General total**	75	63	**83.9**	12	**16.1**
1.1.2 SUPPRESS	2	2	100	0	0.0
1.1.4 INACTIVATE	15	11	73.3	4	26.7
1.4.0 MODIFY	8	6	75	2	25
2.3.0 INTERACT	21	19	90.5	2	9.5
4.1.3 LABEL	15	11	73.3	4	26.7
8.3.1 TRANSPORT	19	17	89.5	2	10.5
11.0.0 RELEASE	11	10	90.9	1	9.1
**Biomedical total**	91	76	**84.6**	15	**15.4**
**Total**	166	139	**84.3**	27	**15.7**

The evaluation shows that over 83% of the new candidates generated across the two domains are valid class members, and in each of the 14 classes individually the majority of novel classifications are correct. From the total number of 166 novel candidates, 139 were judged as correct, which demonstrates that our automatic method can be used as a highly accurate starting point for the creation of BioVerbNet.

In two of the evaluated classes, ’Conduct’ in the general domain and ’Suppress’ in the biomedical domain, all of the novel classifications were marked as valid member verbs, while in four other classes - ’Examine’ and ’Indicate’ in the general domain and ’Interact’ and ’Release’ in the biomedical domain - over 90% of new candidates were judged as correct. The ’Conduct’ class provides a good example of how our system accurately selects candidates that are semantically similar to the existing class members based on similarity of their syntactic behaviour: the original member verbs, *perform* and *conduct*, are provided with new synonymous counterparts, *execute* and *undertake*. Analogous cases are found in the biomedical domain, e.g., in the ’Interact’ class, a new candidate *collaborate* is a close synonym of one of the original class members, *cooperate*. What is more, our classifier picks up not only synonymous, but also antonymous verbs as candidates for a given class, as seen in the biomedical domain (e.g. *downregulate - transactivate*). This is consistent with what has been observed in previous work on manual semantic classification of verbs [52], where human annotators were found to consistently group antonyms together as semantically similar. Despite representing the opposites of a meaning continuum, antonyms have almost identical distributions, and that paradigmatic similarity is what makes annotators judge them as semantically closely related.

An in-depth analysis of the candidate verbs by the experts sheds light on the strengths of the presented approach, as well as the error patterns and areas for future improvement. Overall, only 15.7% of new candidates were judged as incorrect across all 14 classes, with slightly more noise found in the general scientific classes (16.1%) than in the biomedical classes (15.4%). In the general language domain, the linguists identified between 0-3 incorrect candidates per class, whereas in the biomedical domain, the experts marked between 0-4 candidates per class as incorrect for the class in question, either judged as mistakes or as candidates for reassignment to another class.

Several recurrent reasons behind the erroneously classified verbs can be identified: (a) accidental syntactic parallels, (b) parsing errors, and (c) clustering loosely related verbs (rather than strictly semantically similar).

a Examples of candidates which ended up in a given class purely through accidental syntactic similarity to the existing members are found, for instance, in the biomedical class ’Transport’. The two incorrect candidates identified, *tailor* and *generalize*, share the syntactic contexts of subj#obj (*The methods generalize earlier approaches...*), subj#prep (*This advantage did not generalize to the visual domain*), and subj#obj#prep (*We also generalize some known results from the real-valued case to the complex-valued one*) with the original class members (e.g. *Highly resistive wires transmit intracardiac electrograms*, *Occasionally these viruses transmit to other mammals*, *GPCRs transmit signals through heterotrimeric G proteins*). In the general scientific domain, examples of coincidentally parallel syntactic behaviour between new and original class members were noted, for instance, in the ’Collect’ class: *decide* and *prioritize*, marked as noisy, share the syntactic contexts of subj#obj (*Future research should prioritize addressing symptoms...*) and obj#prep (*Should the surgeon decide on relaparoscopy...*) with *harvest, select* and *collect*.
b In some cases the syntactic contexts themselves were mistakenly identified as identical due to a parser error, which produced noisy candidates. For example, the verb *lie* got classified with the ’Appear’ class members based, among others, on the shared subj#obj#prep context, exemplified by the phrase: *Thermal imaging as a lie detection tool at airports*, where ’lie’ is a noun modifier of ’detection’, both of which form a compound modifying the noun ’tool’, rather than being a verb taking a noun object and a preposition. Or similarly, in the context subj#obj#prep *We review the technical challenges that lie ahead*, ’ahead’ is mistakenly analyzed as the object rather than a preposition. Another type of error had to do with analyzing the particle ’to’ as a preposition rather than an infinitive marker, as in the few cases of misidentified syntactic contexts such as *HIV and HCV seem to co-opt DDX3* as identical to subj#prep: *many interventions may vary between population groups*, or *(...) await for clinical applications*, which contributed to clustering dissimilar verbs such as *vary, await, pave* together in the ’Appear’ class with *appear* and *seem*.
c Another type of misclassification involves candidate verbs which are related to the existing class members but are dissimilar to them with respect to some meaning components or semantic properties identified as characteristic of the class in question. In the biomedical domain, examples of this kind of error are found in the ’Modify’ class, where 8 new candidates are added: *hydroxylate, hydrolyze, methylate, deaminate, esterify, oxidize, detoxify, metabolize*. Out of these, the last two (*detoxify, metabolize*) were flagged as standing out from the rest, based on the fact that they describe processes on the cellular level, in contrast to the rest of member verbs referring to a specific chemical changing (i.e. terms pertaining to the chemical level). In the general scientific domain, examples of related but not strictly similar verbs added through looser association with the existing members include *optimize* and *understand* yielded for the ’Examine’ class, or *cultivate* in the ’Collect’ class.

The new verbs judged as not valid were marked as candidates for reassignment to another existing class, or as members of a subclass or a new class altogether. An incidence matrix showing the class reassignments is presented in Additional file 2 for reference. For instance, *exacerbate, aggravate* and *magnify*, found in the ’Inactivate’ class, were highlighted as forming a separate cluster of similar verbs, while the verb *deacylate* found in the ’Release’ class was reassigned to the ’Modify’ class. In the general scientific domain, an example of reassignment involved verbs *display* and *exhibit*, found in the ’Encompass’ class but considered better suited for the ’Indicate’ class, within which four other candidates, *underline, underscore, highlight, emphasize*, were marked as forming a subclass of ’underline’-type verbs. Such cases demonstrate the potential of the classification method for also discovering valid novel classes not in the original classification.

### Discussion

The in-depth scrutiny of the new candidates shows that our automatic classification approach is highly accurate and thus likely to be very useful for extending the manual classification of biomedical verbs to a large-scale BioVerbnet. Although some human validation and filtering of the noise is necessary for the development of a fully accurate resource, the time and cost required for this is likely to be small in comparison with a fully manual development of such a large resource from scratch. The manual development of the original Levin classification [57] and VerbNet [7] required years of research effort, although semi-automatic methods were used to facilitate their extensions too [58]. Our qualitative analysis shows that despite being based purely on syntactic behaviour and combinatorial properties of verbs, the method also associates verbs in terms of their shared semantics, yielding classes of semantically similar and closely related members.

The error analysis reveals some areas of potential improvement. While the accidental syntactic parallels are a difficult problem to deal with (and have, in fact, been reported to challenge verb classification regardless of the clustering approach adopted [37]), errors from parsing could be addressed in the future via use of tools capable of dealing with the problem cases highlighted in the previous section. Misclassifications involving candidate verbs which are related to the existing class members but dissimilar to them with respect to some semantic properties are not necessarily an issue that needs to be addressed. Rather, such cases may actually demonstrate the potential of the method to hypothesize novel classes and classifications for human validation and offer the means for subsequent refinement of the original classification. This is important because the original classification is, by no means, comprehensive and is likely to require further development as we scale it up to cover language in biomedicine.

## Conclusion

This paper introduces and evaluates an automatic verb classification approach to facilitate cost-effective development of BioVerbNet. From the methodological point of view, our work constitutes the first effort applying a state-of-the-art architecture for neural representation learning to biomedical verb classification. In terms of our contribution to representation learning, while previous works have shown that such neural models can be highly efficient for learning linguistic properties from large corpora, there has been little work on fine-tuning the models for class-specific tasks (e.g. verbs). Our work demonstrates that the learning of class-specific representation is highly context-sensitive. In particular, we identify the contexts that are essential for training representation for biomedical verbs. This can facilitate the development of different learning approaches for class-specific representations as well as support researchers in biomedicine to better understand the syntactic and semantic properties of verbs in biomedical texts.

As a verb classification method, our method is attractive in terms of avoiding the heavy feature engineering involved in most previous approaches. Our evaluation reveals that it is also highly accurate, suggesting that the classification of 957 new verbs created by our method (details in “Automatic verb classification” section) and released with this article could be used to readily support application tasks in biomedicine. Our plan is to ultimately use it to support the development of BioVerbNet via expert validation - an approach that can yield a fully accurate computational resource and enriched taxonomy with novel classes. Such a BioVerbNet, once developed, will provide a welcome addition to lexical resources in biomedicine which largely focus on nouns (e.g. the UMLS Metathesaurus mainly covers noun concepts) or a limited set of verbs (e.g. the BioLexicon provides the syntactic and semantic information of 168 verbs commonly used in E.Coli).

### Future work

The methodology introduced in this paper can be improved further in a variety of ways. First, in the current work we use a supervised approach for verb classification. While this provides an immediate benefit in terms of the accuracy of verbs classified, it requires a fixed set of pre-defined verb classes as part of the training data. To allow unsupervised discovery of novel verb classes and subclasses, one idea for future work would be to improve the performance of unsupervised clustering algorithms with a small amount of supervision in the form of labels on the data (seeds), constraints or user feedback. This type of approach, commonly known as semi-supervised clustering, can not only group candidates using the classes learned from the seed data, but also extend and modify the existing set of classes as needed to reflect other regularities in the data. Studies of this nature are emerging [59, 60] and it would be interesting to investigate how they could be applied to our task to reduce the need for pre-defined classes while maintaining promising precision.

Another potential research avenue is to improve the quality of representation learning through context modelling. Here, our work experiments with dependency-based contexts, showing that they are effective in producing large semantically meaningful groups of classes. Nevertheless, there are a few cases where semantically dissimilar verbs are mis-classified together because they share similar syntax, showing room for improvements in identifying other discriminative contexts.

Last but not least, our representation models are trained on word co-occurrence frequencies to capture verb semantics on the word-level. Because many word formations in biomedicine follow rules (e.g. *phosphorylate* and *dephosphorylate*), it is possible to improve representation learning by incorporating both word and character-level information. In future, we plan to explore other representation learning techniques for verb classification including FastText [61], whose learning procedure takes into account the morphological (subword) information. To encourage future research in related aspects, we make our resource available to the community at https://github.com/cambridgeltl/bio-verbnet.

## Additional files

Additional file 1 Annotation guidelines. This document includes the guidelines used in human validation of verb classes reported in the paper. (PDF 179 kb).

Additional file 2 Supplementary Information. (PDF 99 kb)

## Abbreviations

BOW: Bag-of-word context
NER: Named entity recognition
NLP: Natural language processing
SGNS: Skip-gram model with negative sampling
