# Supplementary material for: A neural classification method for supporting the creation of BioVerbNet
Source: J Biomed Semantics. 2019 Jan 18;10:2. doi: 10.1186/s13326-018-0193-x (PMC6339329; doi:10.1186/s13326-018-0193-x)
Supplement: Supplementary file 1 — Annotation guidelines. This document includes the guidelines used in human validation of verb classes reported in the paper. (PDF 179 kb). [file 13326_2018_193_MOESM1_ESM.pdf]

Contact Person: Billy Chiu
Email: hwc25@cam.ac.uk

Guidelines for Classification of Biomedical Verbs

1 Background

The experiment aims to extend the small biomedical verb classification of Korhonen et al. (2006) [1] with the view facilitating the creation of BioVerbNet. The small classification contains 192 verbs organized into a 3-level taxonomy consisting of 16, 34 and 50 classes. We have now applied an automatic classification approach (described in the associated paper draft) to create an extended classification. It consists of 1,149 verbs in total (the 192 original ones plus 957 new ones) that have been grouped into the original class taxonomy based on their shared meanings and syntax according to our learning technique.

Your task is to verify whether these new candidate verbs are really similar in terms of their meanings as well as syntactic patterns to existing verbs in the original classification. Here is our initial proposal for how the task could be conducted.

The task has the following the steps (in blue, tasks to be answered in the Excel spreadsheet: **Answer.xlsx**):

2 Task A: Decide whether new verbs in each verb class share the similar meanings and syntactic patterns

2.1 Materials

You will be provided with 3 documents to support this task. They are:

1. **Question.xlsx**: The list of verbs grouped into classes, with descriptions of each class.
2. **Answer.xlsx**: An Excel spreadsheet for recording the updated index of the class for each verb based on your perception.
3. **Examples** (folder): Example sentences for each verb.

Please download and unzip the materials from:
`https://drive.google.com/open?id=1tbV92X7fG13ereSIxo2oTC0Ly1zGbXqM`

2.2 Task Description

Open the file: **Question.xlsx**, you will see verbs grouped into classes based on their shared meanings and syntax. They are organised in five columns (see Figure 1) as follows:

Class Name: The name of each class.

Sub-class Name: The name of each sub-class.

Class index: The unique identifier (which you will need to use throughout the entire task).

Example Verbs: Example verbs for each class from the original 192 classification.

New candidates: The list of new candidate verbs for verification.

A	B	C	D	E
**Class Name**	**Sub-class Name**	**Class Index**	**Example Verbs**	**New candidates**
Biochemical events	Express	2.1.0	express overexpress	coexpress,secrete
	Biochemical modification	2.2.1	dephosphorylate phosphorylate	deacetylate,ubiquitinate,acetylate
	Cleave	2.2.2	cleave	
	Interact	2.3.0	react interact interfere cooperate colocalize coincide correlate	cosegregate,compensate,collaborate,participate,coevolve,correspond

Figure 1: A screen-shot of the subset of verb class in *Question.xlsx*. *Class Name* is the name of the top-level class. *Sub-class Name* is the name of each sub-class. *Class index* is the unique identifier of each class/sub-class. *Example Verbs* has the member verbs of each sub-class. *New candidates* contains verbs to be verified by annotators. They are separated from *Example verbs* by red line for distinction

.

Your task is to decide whether each new candidate verb (i.e. *New Candidates* in Fig 1) has been assigned to the right class/sub-class based on your interpretation of the *Example verbs* in each class, as well as the sentence examples we provided for each verb (in the **Example** folder, as describes in Section 2.2.1). You should give your answers on the file we provided (**Answer.xlsx**, as describes in Section 2.2.2).

2.2.1 Sentence Examples

To help you understand how a verb is used in biomedical text, we provide about thirty example sentences from the corpus we used in our experiment, which illustrate the most common syntactic structures of each verb (in descending order, most common on top and least common at bottom). They are stored in folder: **Example** with the test verb as the filename. They are organized in 3 columns: The first column is the name of the dependency pattern exemplified in the sentence. The second column is the sentence example. The third column is the word in sentences corresponding to the syntactic pattern (see Figure 2).

```
increase.txt
obj This seemed to increase illness-related strain and a need for defensive actions .   obj@strain
obj Disruption of yqhC offers a useful approach to increase furfural tolerance in bacteria .    obj@tolerance
obj Breast feeding does not appear to increase the risk of postpartum relapses .    obj@risk

subj#obj    Inappropriate use of emergency care services can increase hospital readmissions and related costs . subj@use obj@readmissions
subj#obj    Simulated altitude did not increase incidence of ECS .  subj@altitude obj@incidence
subj#obj    However , pharmacological PPARdelta activation did not increase T-cadherin expression . subj@activation obj@expression
```

Figure 2: A screen-shot of example sentences of *increase* (in Folder: *Example*). The first column contains common syntactic patterns for *increase* in descending order (e.g. *obj*=object). The second column stores the sentence example for using the corresponding pattern. The third column stores the corresponding words in the sentence for the pattern (e.g. *strain*)

Look into the sentence examples of each *New candidates* and *Example Verbs* in each class (as mentioned in Section 2.2), decide if each new candidate verb has been assigned to the right class. Give your answers on our answer template in the pre-defined format, which is described in the next section.

2.2.2 Answers Template

Open the file: **Answer.xlsx**, you will see all the new candidates (Column 1) and the classes they are currently assigned to (Column 2). Please write down the *Class Index* (reference from

New candidates	Current Class	Final Class
demethylate	1.1.1	
biotinylate	8.1.0	

Figure 3: A screen-shot of the answer sheet for annotators (filename: *Answer.xlsx*). *New candidates* contains verbs to be verified by annotators. *Current Class* is the index of the class where a verb currently assigned to. *Final Class* records the updated class indexes for the verbs after verified by annotators.

Question.xlsx) you think each verb should be assigned to. Here, we use *demethylate* and *biotinylate* as examples (see Fig 3), they are currently assigned to the Class *1.1.1* and Class *8.1.0* correspondingly. There are three options to choose:

1. If you think the verbs are correctly assigned, just put down the same class indexes as their suggested ones (i.e. *1.1.1* and *8.1.0*) in their corresponding cell in the *Final Class* column.

2. In contrast, If you think the verbs are incorrectly assigned:

   (a) If the mis-assigned verb should be in another class, please put down the corresponding class index. For example, if you think *demethylate* should be in the class: *Biochemical modification* (see Fig 1), then put down its index: *2.2.1* in the *Final Class* column.

   (b) If at least two mis-assigned verbs can be part of an entirely new top class, please put down a new class index in the format: (*N+1.0.0*) where *N* is the current largest top-class index (By default, we have 16 top-level classes (*N=16*), so new index begins with *17.0.0*). For example, if you think *demethylate* and *biotinylate* can be part of an entirely new classes, and this is the first class index you create, then put down *17.0.0* in both of their cells in the *Final Class* column. Subsequent new class index will then be *18.0.0*, *19.0.0*...etc).

   (c) Any verbs you cannot find a good class for, please put in 0 as its class index in the *Final Class* column.

Give a final class index to each new candidate verb. HOWEVER, A VERB CAN ONLY BE ASSIGNED TO ONE CLASS/SUBCLASS ONLY!!!

3 Submission

There is not necessarily a fully correct solution and a perfect grouping to the task. It is perfectly reasonable to use your intuition or gut feeling as a biologist while working on this task. Upon finish, please email back your completed **Answer.xlsx** to Billy at hwc25@cam.ac.uk. Thank you very much for your help.

References

[1] Anna Korhonen, Yuval Krymolowski, and Nigel Collier. 2006. *Automatic Classification of Verbs in Biomedical Texts.*. In Proceedings of ACL-COLING 2006. Sydney, Australia.
