# Supplementary material for: A neural classification method for supporting the creation of BioVerbNet
Source: J Biomed Semantics. 2019 Jan 18;10:2. doi: 10.1186/s13326-018-0193-x (PMC6339329; doi:10.1186/s13326-018-0193-x)
Supplement: Supplementary file 2 — Supplementary Information. (PDF 99 kb) [file 13326_2018_193_MOESM2_ESM.pdf]

Supplementary Information: A Neural Classification Method for Supporting the Creation of BioVerbNet

Billy Chiu, Olga Majewska, Sampo Pyysalo, Laura Wey,
Ulla Stenius, Anna Korhonen and Martha Palmer

2018
December

This document contains supplementary information for the paper: *A Neural Classification Method for Supporting the Creation of BioVerbNet.*

1 Evaluation results for Bag of Word (BOW) representation of different window sizes

In Section 5.1 (Table 2) of our paper, we took the representation models trained with different verb-related contexts and compared them against a generic BOW model with window=5. Here, we provide the BOW results with different window sizes for reference (Table 1).

Window Size	Bio-SimVerb(ρ)
1	0.4694
2	0.4696
4	0.465
5	0.4664
8	0.462
16	0.466
20	0.4524
25	0.4509
30	0.4402

Table 1: Performance on BioSimVerb (in Spearman's ρ) using biomedical representation models trained using different window sizes. We reported the result (win=5) in the paper for brevity.

2 An incidence matrix showing the class reassignments of verbs in BioVerbNet

The new verbs judged as not valid were marked as candidates for reassignment to another existing class, or as members of a subclass or a new class altogether. An incidence matrix showing the class reassignments is presented in Table 2. For instance, *exacerbate, aggravate* and *magnify*, found in the Inactivate class, were highlighted as forming a separate cluster of similar verbs, while the verb *deacylate* found in the Release class was reassigned to the Modify class. In the general scientific domain, an example of reassignment involved verbs *display* and *exhibit*, found in the Encompass class but considered better suited for the Indicate class, within which four other candidates, *underline, underscore, highlight, emphasize*, were marked as forming a subclass of underline-type verbs. Such cases demonstrate the potential of the classification method for also discovering valid novel classes not in the original classification.

	7.1.0 COLLECT	9.1.1 EXAMINE	9.3.0 INDICATE	10.1.3 CONDUCT	13.1.0 ENCOMPASS	14.0.0 CALL	16.0.0 APPEAR	1.1.2 SUPRESS	1.1.4 INACTIVATE	1.4.0 MODIFY	2.3.0 INTERACT	4.1.3 LABEL	8.3.1 TRANSPORT	11.0.0 RELEASE	NEW CLASS	**Counts**
7.1.0 COLLECT													transfuse			1
9.1.1 EXAMINE																0
9.3.0 INDICATE																0
10.1.3 CONDUCT																0
13.1.0 ENCOMPASS			display, exhibit													2
14.0.0 CALL																0
16.0.0 APPEAR																0
1.1.2 SUPRESS																0
1.1.4 INACTIVATE															exacerbate, aggravate, magnify	3
1.4.0 MODIFY															detoxify, metabolize	2
2.3.0 INTERACT																0
4.1.3 LABEL																0
8.3.1 TRANSPORT																0
11.0.0 RELEASE										deacylate						1

Table 2: An incidence matrix showing the class reassignments of verbs in BioVerbNet. It shows how verbs are reassigned from their original classes (rows) to their final classes (columns) as determined by human annotators. **Counts** refer to the total numbers of reassignments of each class. If the annotators cannot find a suitable class to fit-in a verb, it will be assigned to **New Class**.
